# New Advances in the Exploration of Esterases with PET and Fluorescent Probes

**DOI:** 10.3390/molecules28176265

**Published:** 2023-08-27

**Authors:** Alba Gil-Rivas, Beatriz de Pascual-Teresa, Irene Ortín, Ana Ramos

**Affiliations:** Departamento de Química y Bioquímica, Facultad de Farmacia, Universidad San Pablo-CEU, CEU Universities, Urbanización Montepríncipe, 28668 Boadilla del Monte, Spain; alba.gilrivas1@ceu.es (A.G.-R.); bpaster@ceu.es (B.d.P.-T.)

**Keywords:** positron emission tomography (PET), fluorescent probes, esterases, carboxiesterases, acetylcholinesterases, butyrylcholinesterases, neurodegenerative diseases, cancer

## Abstract

Esterases are hydrolases that catalyze the hydrolysis of esters into the corresponding acids and alcohols. The development of fluorescent probes for detecting esterases is of great importance due to their wide spectrum of biological and industrial applications. These probes can provide a rapid and sensitive method for detecting the presence and activity of esterases in various samples, including biological fluids, food products, and environmental samples. Fluorescent probes can also be used for monitoring the effects of drugs and environmental toxins on esterase activity, as well as to study the functions and mechanisms of these enzymes in several biological systems. Additionally, fluorescent probes can be designed to selectively target specific types of esterases, such as those found in pathogenic bacteria or cancer cells. In this review, we summarize the recent fluorescent probes described for the visualization of cell viability and some applications for in vivo imaging. On the other hand, positron emission tomography (PET) is a nuclear-based molecular imaging modality of great value for studying the activity of enzymes in vivo. We provide some examples of PET probes for imaging acetylcholinesterases and butyrylcholinesterases in the brain, which are valuable tools for diagnosing dementia and monitoring the effects of anticholinergic drugs on the central nervous system.

## 1. Introduction

Several vital physiological processes in living organisms are regulated by enzymes, which act as highly efficient biocatalysts. Enzymes are classified into six major classes based on the type of reaction that they catalyze: oxidoreductases, transferases, hydrolases, lyases, isomerases, and ligases [1]. Among these, esterases, a type of serine hydrolase, hold significant importance and find wide applications in biotechnology, pharmaceuticals, environmental sciences, and various industrial fields [2]. In the human body, esterases are predominantly expressed in organs and tissues that have barrier functions, such as the lungs, small intestine, liver, kidney, skin, and even the brain [3,4]. These enzymes catalyze biotransformations by hydrolyzing ester-containing compounds, leading to the formation of the corresponding acids and alcohols. They play essential roles in regulating metabolism, gene expression, signal transmission and transduction modulation, detoxification of xenobiotics, and bioactivation of prodrugs [5,6]. Dysregulation of esterase homeostasis is associated with various pathological conditions, including cancer, obesity, Wolman disease, or neurodegenerative disorders [7,8,9].

Monitoring the activity of these enzymes has gained significant importance in the scientific community due to its potential to provide crucial physiological information and contribute to the early and accurate diagnosis of numerous diseases [10,11,12]. This task has been facilitated by the development of new imaging non-invasive in vivo visualization approaches [13] with broad applicability, operating at various levels including molecular, cellular, organ, and even whole organism [13,14].

Well-established imaging strategies encompass a range of techniques, including non-radioactive methods such as optical fluorescence as well as radioactive PET methods, which are the most widely used. Both approaches will be the focus of this review for the development of new esterase probes for in vitro and in vivo bioimaging applications. It is important to emphasize that the design of suitable probes must consider diverse spatiotemporal requirements, depending on the specific scope of the study, which can range from investigating single cells to examining the entire body. Key considerations in probe design include selectivity, depth of tissue penetration, resolution, sensitivity, pharmacokinetic profile, production cost, and other relevant factors [11].

## 2. Optical Fluorescence Technique and Fluorescent Probes

**Figure 1:**
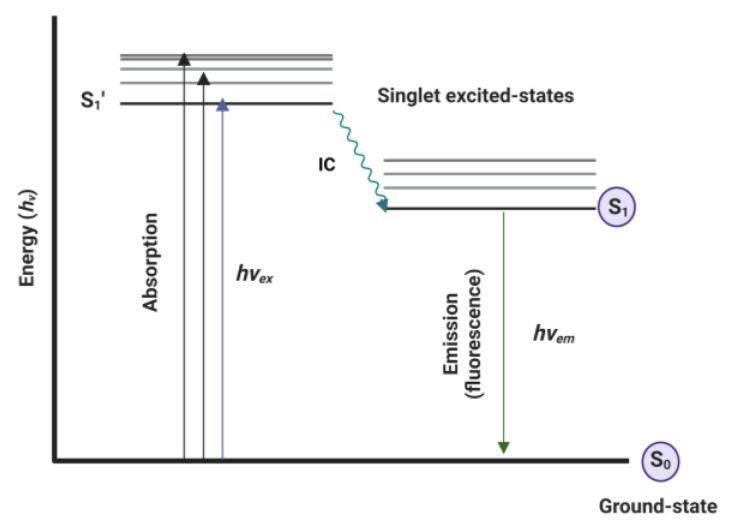
Jablonski diagram illustrating the process of excitation and emission of fluorescence [16]. Image created using BioRender (www.biorender.com (accessed 23 August 2023)).

Optical fluorescence is a non-radioactive technique that involves the administration of a fluorescent agent. This agent is excited (hv_EX_) by an external light source, passing from the ground state (S_0_) to the excited-state life (S_1_′). This state evolves to the S1 excitation state by internal conversion (IC), which immediately triggers emission (hv_EM_) with lower energy and a greater wavelength than the excitation light source. During this phenomenon, some of the energy is dissipated as heat or through other non-radioactive processes to S_0_ or intersystem crossing (ISC) [15]. This process is illustrated by the Jablonski diagram shown in Figure 1 [16].

Fluorescent probes, when combined with the widespread use of confocal microscopy, have demonstrated several advantages over traditional modalities such as computed tomography (CT), magnetic resonance imaging (MRI), or radioisotope imaging. These advantages include ease of use, high sensitivity and resolution, low cost, and non-invasive real-time detection [1,11].

Most probes consist of three scaffolds: a fluorescent moiety that serves as the signaling component and is responsible for the spectroscopic properties; a recognition moiety that acts as the binding component to the target; and a linker that connects both fragments [1]. The fluorescent component incorporated into the probe can exist in two forms: agents that exhibit fluorescence upon excitation, known as fluorophores, and those that require prior modification to acquire fluorescent properties, known as fluorogenics. This latter modification can occur after binding to a target [17], through a chemical reaction [18], or even due to alterations in their environment, such as changes in pH, hypoxia, the presence of reactive oxygen or sulfur species, or the presence of metal ions [15,19,20].

The applicability of fluorescent probes depends on changes in fluorescence properties such as emission intensity, wavelength, and lifetime. Therefore, their design is aimed at achieving accurate signal modulation and adequate selectivity towards the target. This design typically involves controlling sensing mechanisms and a wide range of fluorophores and fluorogenic agents.

The fluorescence emission of most fluorescent probes relies on various sensing mechanisms, including: photoinduced electron transfer (PET) (I); intramolecular internal charge transfer (ICT) (II); Förster resonance energy transfer (FRET) (III), excited state intramolecular proton transfer (ESIPT) (IV); aggregation-induced emission (AIE) (V); and multiple modality fluorescence approaches, which are widely described in the literature [21,22].

**Figure 2:**
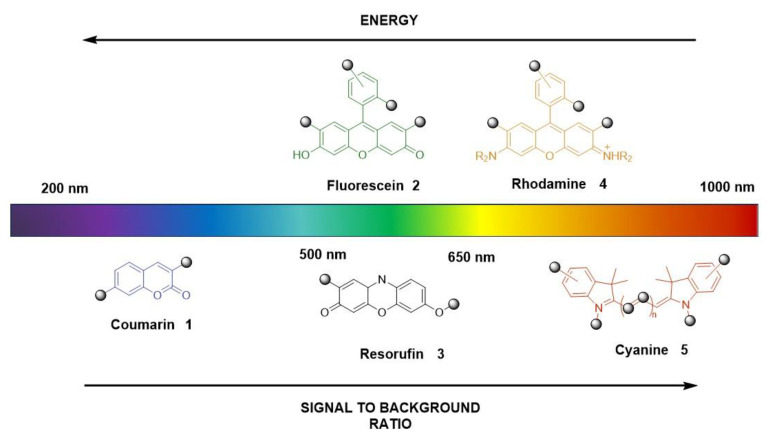
Summary of most common fluorophores by structure and emission color [23].

The choice of fluorophores or their combination can span from the ultraviolet-visible (200–600 nm) to the near-infrared or far-red spectral regions (650–1000 nm), which determines the excitation/emission profiles (Figure 2). 

**Figure 3:**
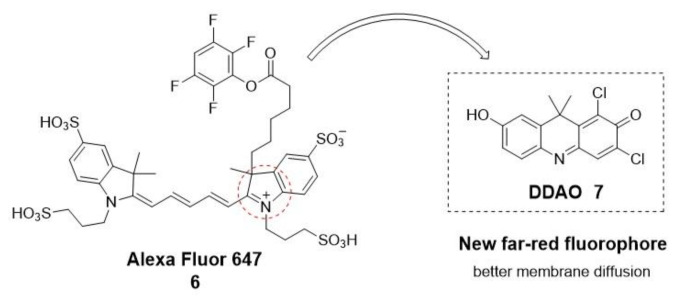
Structure of two types of far-red fluorophores.

Over the past 10 years, most existing fluorescent probes for esterases have been based on fluorophores such as coumarin (**1**), fluorescein (**2**), resorufin (**3**), and rhodamine (**4**) [24]. However, their limited ability to penetrate tissues, light scattering, and interference from background auto-fluorescence issues have led to the increased use of near-infrared and far-red fluorescent probes for bioimaging applications [23]. Among these cyanines, such as Alexa Fluor 647 (**6**), Cy5, and Cy7, many are widely applied. Nevertheless, one main limitation is their difficulty in passing across cell membranes due to their positive charge. As an alternative to these far-red cyanines, 7-hydroxy-9*H*-(1,3-dichloro-9,9-dimethylacridin-2-one) (DDAO, **7**) has been reported (Figure 3) [25]. 

As an alternative to overcoming the limitations of fluorescent probes based on fluorophore scaffolds with an “on-off” approach, researchers have explored other strategies. These approaches involve the use of fluorogenic moieties and leverage various response modes in an “off-on” fashion. The “off-on” approaches have been efficiently designed to function as enzyme substrates [1]. 

**Scheme 1:**
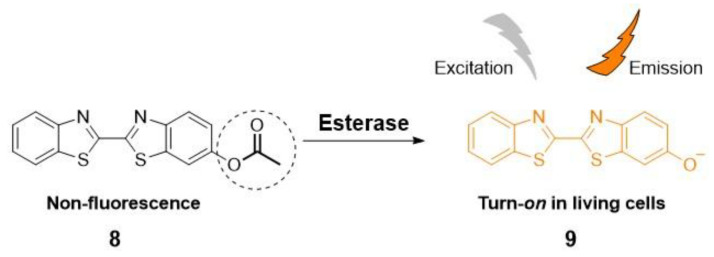
Turn-on fluorescent probe **9** by esterase hydrolysis.

An “off-on” type of fluorescent probe, designed to modify the fluorescence by an enzymatic response, can be prepared, for example, by masking a functional group (e.g., free -OH or -NH_2_) on the fluorophore with an enzyme recognition moiety (e.g., acetoxy or acetoxymethyl ether), as illustrated in Scheme 1 [26]. In this way, the fluorescence is quenched until the functional group is deprotected by the enzymatic action. 

**Figure 4:**
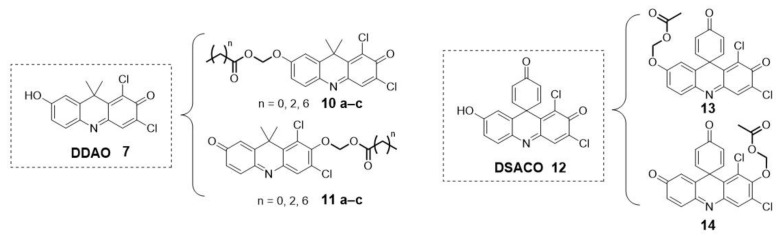
Examples of far-red fluorophores with an esterase masking group.

Some additional examples include derivatives of the previously mentioned DDAO (**7**) or a subclass of spiro-type derivatives (DSACO, **12**) with an esterase-masking moiety (Figure 4) [25,27]. These probes have been used for detecting and tracking esterase and lipase activities and profiling esterases of *Mycobacterium* species [25,28].

**Scheme 2:**
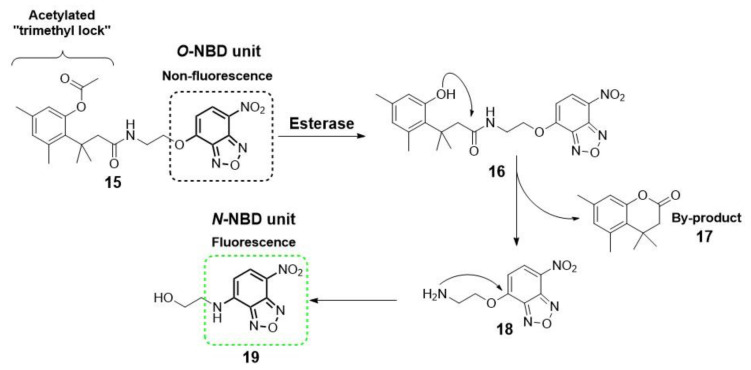
Conjugate of NBD and self-immolative linker based on trimethyl lock for the detection of esterases [30].

Another alternative for designing an “off-on” fluorescent probe is the use of a self-immolating linker (e.g., a *p*-hydroxybenzyl alcohol unit and an acetylated “trimethyl lock” system) to connect both the fluorogenic and the recognition moieties [1]. Upon enzymatic transformation, the structural change triggers spontaneous cleavage of the linker unit, releasing the fluorophore [29]. Okada and collaborators describe a representative example, illustrating the utility of this self-immolative linker for the detection of esterase activity by conjugation of an acetylated “trimethyl lock” and a nitrobenzoxadiazole (NBD, **19**) unit (Scheme 2) [30]. 

**Scheme 3:**
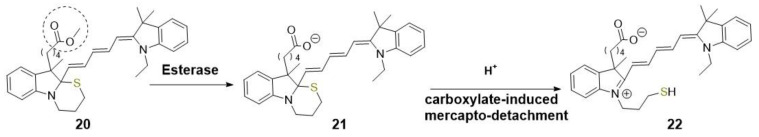
Enzyme-mediated turn-on mechanism in which the quenched fluorophore is activated (turned on) through activator-induced quencher-detachment.

The key advantage of this strategy lies in reducing the steric hindrance between the bulky fluorophore and the enzyme active site, thereby enhancing the efficiency of the probe as an enzyme substrate. However, a potential concern arises due to the release of by-products during the self-immolation process, which could have adverse effects on living systems. To address this issue, researchers have introduced a new class of fluorescent probes comprising two main units: a fluorescence quencher and an activator, employing a turn-on mechanism (as depicted in Scheme 3). When the esterase initiates the cleavage of the carboxylate (quenched form, **21**), it acts as a fluorescence activator, leading to the detachment of the mercapto group (quencher). As a result, the emissive cyanine-based fluorophore Cy5 (**22**) is generated without the formation of any by-products. This approach ensures a more controlled and safer activation of the fluorophore, making it a promising solution for biological applications [31].

**Scheme 4:**
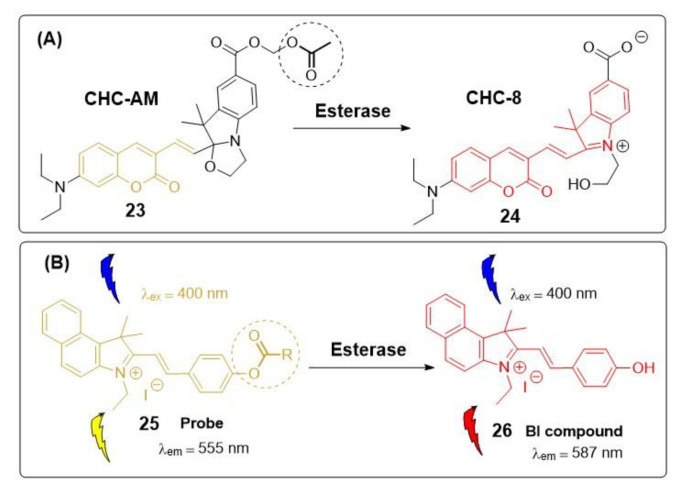
Illustration of two examples of ratiometric fluorescent probes for imaging esterases. (**A**) Illustration of the mechanism of probe **23** response to esterase [32]. (**B**) Illustration of the mechanism of probe **25** response to esterase in HEPES buffer [33].

As a combination of both methodologies (“on-off” and “off-on”), ratiometric probes arise. These probes present an attractive and reliable alternative for the development of endogenous esterase detection tools. In this approach, a ratiometric fluorophore binds to the recognition moiety, as illustrated in Scheme 4 [32,33]. After the hydrolysis reaction by the esterase, there is a noticeable shift in either the emission or excitation wavelength. This shift enables the calculation of the ratio between the fluorescence intensities at the two wavelengths. As a result, the accuracy and sensitivity of detection are significantly improved, and potential interferences, such as instrumental conditions, are eliminated. Moreover, these ratiometric probes possess a self-calibration capability, setting them apart from the “off-on” probes discussed earlier. Compared to the “on-off” approach, ratiometric and “off-on” approaches are generally preferred because they offer several advantages, including reduced background signal [1]. These strategies enhance the performance of esterase detection tools, making them more versatile and reliable for various biological applications.

Recent research has presented illustrative examples of fluorescent probes designed to target esterases. These probes utilize the aforesaid sensing mechanisms to enable efficient and selective detection of esterase activity.

### 2.1. Imaging and Therapy: Fluorescent Probes for Esterases

#### 2.1.1. Cells Viability

The intrinsic machinery of cells encompasses a series of physiological and pathological processes, including apoptosis and necrosis, aimed at eliminating unwanted cells and ensuring cellular viability. During cell death, several changes take place, and one of the most notable is the loss of esterase activity levels [34]. 

**Scheme 5:**
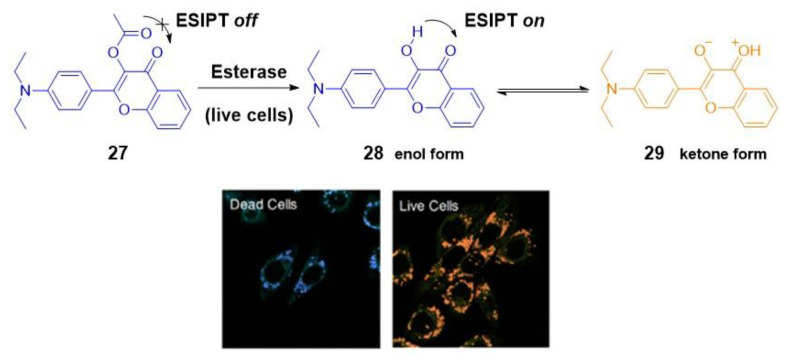
Sensing mechanism based on ESIPT process of fluorescent probe **27** for discriminating live (orange) and dead cells (blue) in dual color mode [34,37]. Reprinted with permission from [37]. Copyright © 2018 American Chemical Society.

Numerous fluorescent probes have been developed to monitor cell characteristics, utilizing some of the previously mentioned sensing mechanisms [35,36]. A representative example was presented by Tian et al. In their study, they devised a ratiometric dual-color fluorescent probe specifically designed to differentiate between live and dead cells based on esterase activity. The core of their strategy involved a chemical modification of the fluorophore 3-hydroxyflavone (**27**). By esterification of the hydroxyl group, the ESIPT process in dead cells was blocked (blue color, 440 nm). In contrast, in live cells, where hydrolysis could occur due to the esterase activity, the ESIPT phenomenon was allowed to occur, yielding an orange color emission at 570 nm (Scheme 5) [37].

**Scheme 6:**
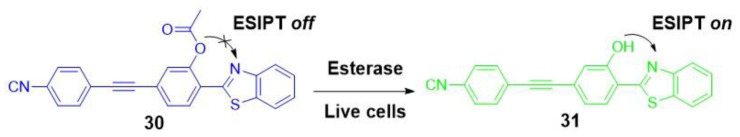
Sensing mechanism of the probe **30** to evaluate cell viability [38].

Another example of an ESIPT-based fluorescent probe intended for assessing differences in esterase activity as a cell viability test was presented by Lu et al. [38]. In their research, they developed a ratiometric probe known as BTE (**30**). This probe exhibited a distinct change in fluorescence emission from short-wavelength blue (at 465 nm) to longer-wavelength green (at 543 nm) in response to esterase hydrolysis within live cells. This transformation was attributed to the cleavage of the acetoxy group present in the probe BTE, caused by esterase-mediated hydrolysis in live cells (Scheme 6).

**Figure 5:**
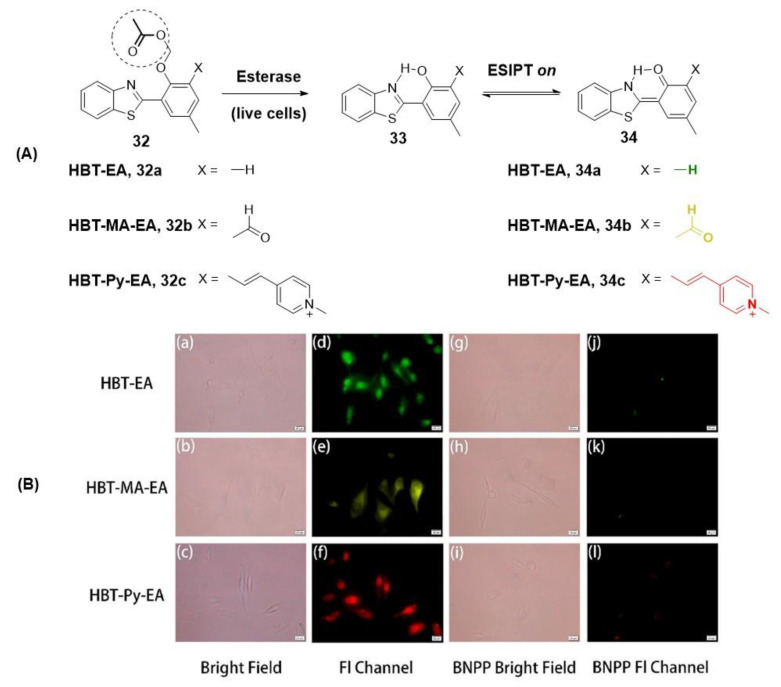
(**A**) Structures and ESIPT response mechanism of probes **34a**–**c** for specific detection of esterase and (**B**) fluorescence microscopy images of HeLa cells incubated with fluorescent esterase probes (**a**–**f**) (**34a** exhibited dark green (**d**), **34b** in yellow (**e**) and **34c** in red (**f**) fluorescence) and bis(4-nitrophenyl) phosphate-BNPP (inhibitor) for intracellular esterase inhibition experiments (**g**–**l**) [5]. Reprinted from publication [5], Copyright 2021, with permission from Elsevier.

Modifications to the structure of the aforementioned fluorophore, based on the common 2-(2′-hydroxyphenyl)-benzothiazole (HBT, **32**), have also been explored by various researchers to detect esterase levels. Notably, Kong and collaborators have achieved the successful synthesis of multicolor fluorescent probes that exhibit a remarkable fluorescence color change upon hydrolysis catalyzed by endogenous esterase in living HeLa cells (Figure 5) [5]. These fluorescent probes have been validated for their use in evaluating the efficacy of esterase inhibitors, showing another valuable application of this imaging strategy.

**Scheme 7:**
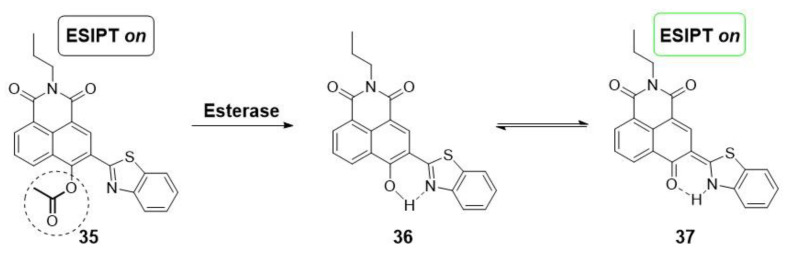
Esterase fluorescent probe based on naphthalimide-benzothiazole conjugation and modulated by the ESIPT mechanism [6].

More recently, a novel acetylated conjugate of naphthalimide and benzothiazole groups (**35**) has been employed for the qualitative detection of esterase inhibition in cells treated with orlistat, an obesity treatment. This innovative fluorescent probe, once again, relies on the activation of the ESIPT sensing mechanism, triggered by the release of a free hydroxyl group induced by esterase hydrolysis. Consequently, this process leads to the emission of fluorescence through enol/keto phototautomerization (Scheme 7) [6].

**Figure 6:**
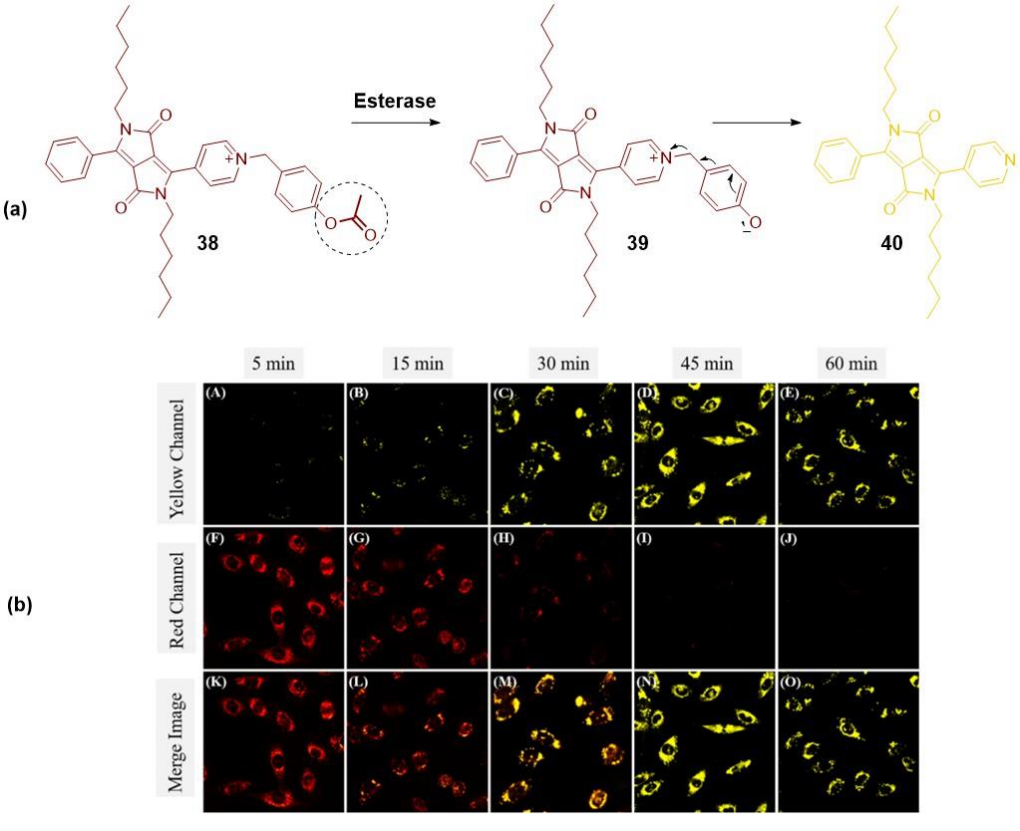
(**a**) Chemical structures of fluorescent probe **38** and sensing mechanisms to detect esterase activity and cell viability. (**b**) Fluorescent imaging of TPC1 cells stained with **38** at different incubation times ((**A**,**F**,**K**) 5 min; (**B**,**G**,**L**) 15 min; (**C**,**H**,**M**) 30 min; (**D**,**I**,**N**) 45 min; (**E**,**J**,**O**) 60 min) in different fluorescent channels ((**A**–**E**) yellow channel (525–585 nm); (**F**,**J**) red channel (625–685 nm) and (**K**–**O**) merge image). λex = 490 nm. Scale bar = 10 μm [34,39]. Reprinted with permission from [39] Copyright © 2018 American Chemical Society.

An appealing approach to enable optical imaging of esterase functions and levels involves the design of ratiometric dual-color fluorescent probes that control the ICT process. Wang et al. adopted this strategy and made modifications to the core of a diketopyrrolopyrrole dye (**38**) to visualize esterase activity and distinguish between live and dead cells. To achieve this, they incorporated a phenyl acetate group on the pyridine, forming a pyridinium cation with a strong red emission (at 655 nm), attributed to the ICT phenomenon. In the presence of active esterase in living cells, the acetoxyl group undergoes cleavage, halting this signaling mechanism and resulting in the emission of fluorescence in the yellow range (at 551 nm) (Figure 6) [39].

#### 2.1.2. Organelles-Targeted Fluorescent Probes

As previously discussed, the design of fluorescent probes can be customized not only for cellular-level exploration but also for targeting organelles such as mitochondria, endoplasmic reticulum-ER, or lysosomes. While there are numerous probes available for cytosolic esterase detection, the development of organelle-targeted fluorescent probes that offer reliable in situ monitoring of esterase activity remains relatively limited.

**Figure 7:**
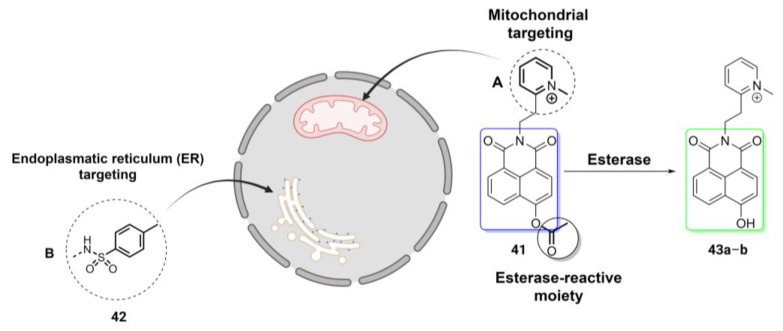
Modification of the target moiety on two different ratiometric fluorescent probes based on 4-hydroxynaphthalimide scaffold and ICT process, for exploring specific organelles [7,40]. Image created using BioRender (www.biorender.com (accessed on 17 August 2023)).

An illustrative demonstration of the adaptability of this type of imaging technique to various targets is seen in ratiometric fluorescent probes based on the 4-hydroxynaphthalimide (NHI) scaffold, as described in two independent studies [7,40]. In both works, Shen et al. and Guo et al. tailored the probes for specific organelles by introducing distinct targeting moieties, as shown in Figure 7. In the study by Shen et al., a methyl pyridinium cation was incorporated into the NHI skeleton as a mitochondria-targeted moiety **41**, whereas Guo et al. used a *p*-toluenesulfonamide group **42** as an ER-directed ligand. In both cases, the acetoxyl group served as the esterase-reactive moiety, inducing changes in the fluorescence emission from the blue to green range. The experimental results from both studies confirmed that these organelle-targeted probes **43a**–**b**, relying on the ICT mechanism, serve as suitable tools for esterase detection.

**Scheme 8:**
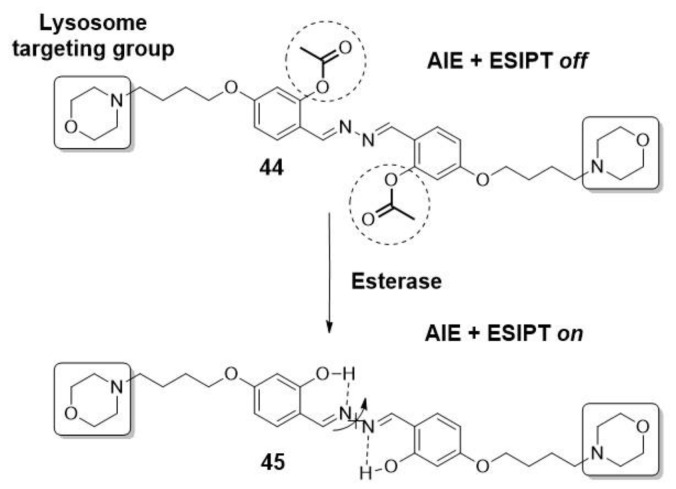
Fluorescent probe for targeting lysosomal esterase based on AIE and ESIPT mechanisms [41].

Gao et al. achieved specific detection of the lysosomal esterase, which is implicated in Wolman disease, using a fluorescent light-up probe. This probe (**44**) was constructed based on a salicyladazine fluorophore bearing esterase-reactive acetoxyl groups and incorporating morpholine as the lysosome targeting moiety (Scheme 8). The main feature of this probe lies in the combination of both AIE and ESIPT mechanisms within a single entity. This combination enhances the sensitivity and efficiency of the probe in detecting lysosomal esterase activity. Moreover, it is noteworthy that the design can be extended for the detection of other enzymes or analytes through the conjugation of different cleavable/recognition elements, allowing for potential applications in various research and diagnostic fields [41].

The unblocking of hydroxyl groups activates the ESIPT process, which is facilitated by the possibility of intramolecular hydrogen bond formation. Additionally, this unblocking promotes AIE by restricting the free rotation of the *N*-*N* bond [41].

**Scheme 9:**
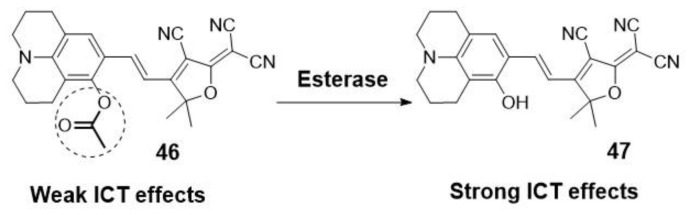
Design of near-infrared fluorescent probe and sensing mechanism for monitoring esterase in tumors [42].

Another fluorescent probe targeting the endoplasmatic reticulum has been recently reported by Xiang et al. They designed a near-infrared fluorescent probe for monitoring esterase changes in tumors in vitro and in vivo. As shown in Scheme 9, they analyzed the esterase-catalyzed hydrolysis by activation of an ICT process in naphatilimide derivative **46**. They based their design on the advantage of the high lipophilicity of the probe to target the endoplasmatic reticulum [42].

**Scheme 10:**
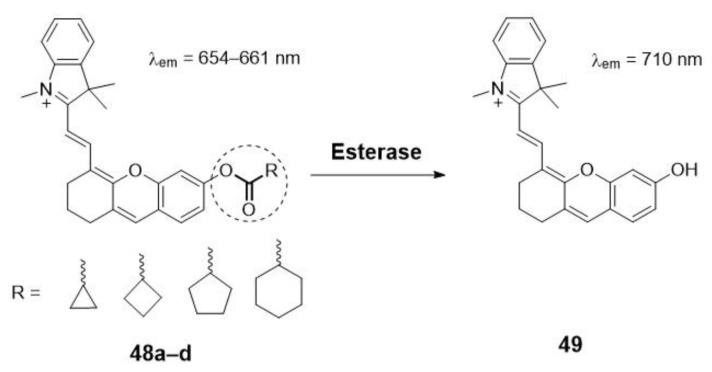
Near-Infrared fluorescent Probes with different ring structures trigger groups targeting mitochondrial esterase [43].

Other authors have also focused on the quantification of esterase activity in mitochondria. For example, Wang et al. designed a new series of ratiometric near-infrared fluorescent probes (**48a**–**d**) for esterase detection (Scheme 10). In this case, the ester hydrolysis of the different ring trigger moieties released the fluorophore **49**. The difference in the change in the plane-twisted dihedral angle deflection in the molecules allows the calculation of the ratiometric fluorescence [43].

**Scheme 11:**
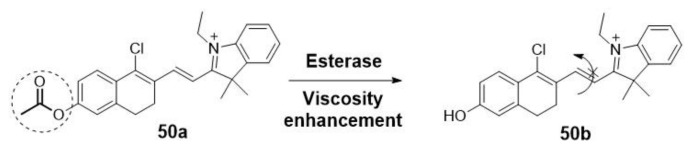
Fluorescence imaging for discriminating early necrosis by detecting changes of mitochondrial viscosity [43].

Lai et al. [43] used the same approach to detect changes in mitochondrial viscosity in living cells associated with early necrosis processes. The presence of a positively charged hemicyanine is the key factor in its accumulation in mitochondria. Moreover, after hydrolysis by the esterase, the twisted intramolecular charge transfer process produces a stronger fluorescence signal (Scheme 11). This phenomenon is especially relevant in early necrotic cells compared to healthy ones. Thus, differentiation among live, death, and necrotic stages was reported.

**Figure 8:**
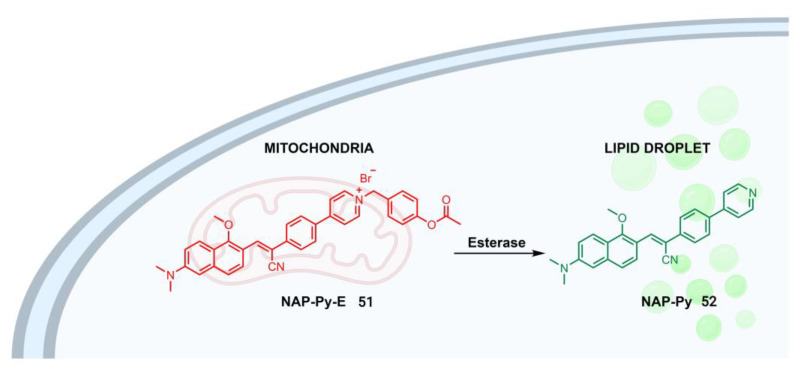
Dual-imaging approach of an esterase-responsive AIE probe for targeting mitochondria and lipid droplet [44,45]. Image created using BioRender (www.biorender.com (accessed on 26 July 2023)).

Recent studies have also focused on the development of fluorescent probes with a dual-imaging approach for studying the relationship among different organelles. This approach enables the detection of complex biological events, providing information about the implications of various organelles with different functions in the same disease. In addition, these multifunctional probes could be used to track drug metabolism. In this regard, an esterase-responsive AIE probe has been designed and synthesized for mitochondrial/lipid droplet dual imaging (Figure 8) [44,45].

A pyridinium cation was introduced in the structure as a mitochondria-targeting group. When the probe NAP-Py-E **51** targets the mitochondria, it interacts electrostatically with electronegative membranes, resulting in the emission of a near-infrared red fluorescence. Then, the acetoxyl group of NAP-Py-E **51** is hydrolyzed by mitochondrial esterases in living cells, converting it to NAP-Py **52**, which reflects esterase activity and cell viability. The specific aggregation of NAP-Py **52** in lipid droplets shows a green emission, leading to a dual-color emission from the two-organelle targets.

**Figure 9:**
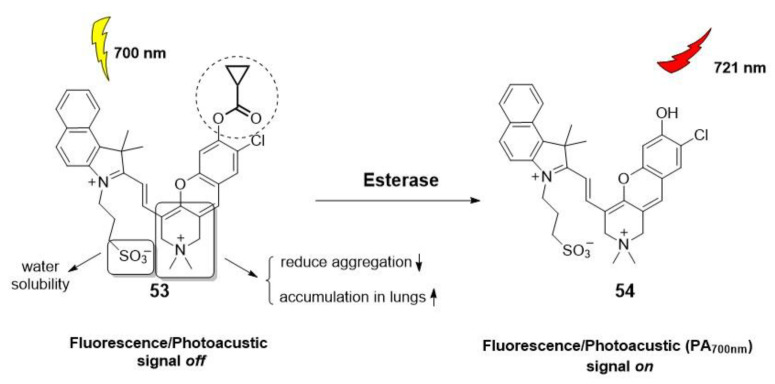
Activatable Photoacoustic/Fluorescent Probes for imaging lung diseases by esterase activation [46].

The scope and versatility of this imaging technique are further demonstrated by its combination with other tools. For example, Fan et al. recently presented an activatable photoacoustic/fluorescent probe, responsive to esterases, for real-time imaging of acute injury (Figure 9) [46]. This probe (**53**), based on a modified structure of the fluorophore hemicyanine, overcomes the limitations of common contrast agents, such as limited retention in pulmonary alveoli, poor blood circulation, and biotoxicity. As a result, this probe offers an effective tool for early diagnosis of the progression of various lung diseases through intravenous injection.

**Scheme 12:**
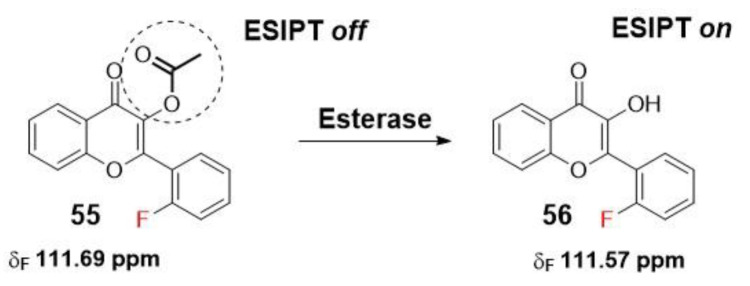
A dual fluorogenic and ^19^F NMR probe for the detection of esterase activity [47].

Another innovative study introduces the use of ^19^F NMR in combination with a fluorogenic flavonoid derivative that includes an esterase-masking moiety. Upon esterase-mediated activation by cleaving the acetoxyl group, the probe undergoes an ESIPT effect, resulting in changes to the ^19^F chemical shifts (Scheme 12). This approach yields a dual-response probe with good sensitivity, selectivity, and real-time detection capabilities, along with an improved imaging depth [47].

**Scheme 13:**
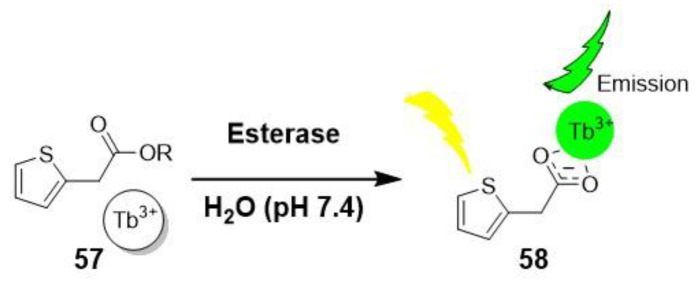
Terbium (III) luminiscense assay for detecting esterase activity [48].

The growing interest in new probes has led to the exploration of rare earth terbium (III) as a potential luminescence assay for detecting esterase activity in the subnanomolar range. These probes, developed by Hetrick et al., were inspired by the photoluminescent signal generated through coordination with effective sensitizers, such as carboxylate groups. This approach extends their use to non *π*-conjugated systems, making them valuable alternatives to common fluorescence probes. As depicted in Scheme 13, esters of thiopheneacetic acid (**57**) are hydrolyzed by the esterase, triggering the sensitization of Tb^3+^ and resulting in luminescence [48]. The use of terbium (III) offers exceptional sensitivity, enabling the detection of esterase activity at remarkably low concentrations.

#### 2.1.3. Fluorescent Probes for Tissues and Organs

In addition, there has been a focus on developing fluorescent probes for specific types of esterases, aiming to achieve selective diagnosis of abnormal functions in tissues and organs. These probes are designed to differentiate among different types of esterases, which may be differentially expressed and have varying substrate specificities. Notably, a significant number of examples described in the literature have focused on probes targeting carboxylesterases (CEs), particularly carboxylesterase 2 (CE2), and only a few examples have used cholinesterases (ChEs). With regard to ChEs, we can highlight several noteworthy probes. Firstly, there is a semisynthetic probe designed based on the SNIFIT concept [49]. Secondly, a boronate fluorescent probe is utilized for the detection of the h202 generation [50]. Lastly, there are probes specifically designed for screening acetylcholinesterase inhibitors [51].

#### 2.1.4. Carboxylesterases

These enzymes belong to the a,b-fold intracellular serine hydrolase family, and the main isoform CE2 exhibits heterogeneous distribution in both healthy and tumor tissues [52,53]. CE2 plays an important role in the phase I metabolism of endobiotics and is also involved in the detoxification/activation of drugs, particularly oral anticancer prodrugs [54]. Therefore, there is a growing need for the design and development of probes for monitoring the activities of these enzymes during pathological disorders, such as different types of cancer, and for screening or evaluating the effects of anticancer drugs. Several examples have been designed based on the aspects mentioned above. Additionally, Dai et al. present a minireview featuring specific examples of carboxylesterase fluorescent probes, providing further insights into the diversity and potential applications of these innovative imaging tools [4].

**Scheme 14:**
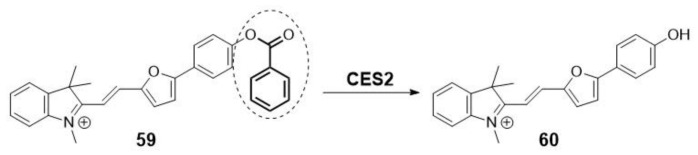
Imaging molecular tool for monitoring CES2 through enzyme-mediated activation of an ICT process.

For instance, recent findings reported by Li et al. unveil a novel mitochondria-targeting near-infrared (NIR) molecular imaging tool for monitoring CES2 during liver-related diseases. As depicted in Scheme 14, the design of probe **59** is inspired by the control of the ICT process through the cleavage of the trigger-benzoyloxy group upon reaction with CES2 [9].

**Scheme 15:**
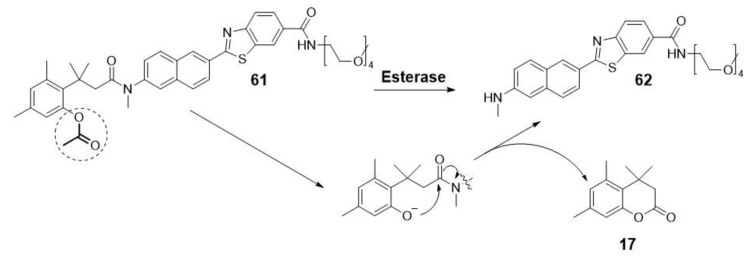
Self-immolative trimethyl-lock linker to design a selective carboxylesterase ratiometric fluorescent probe.

In 2018, Park et al. conducted a sensing study using a ratiometric two-photon probe for CES2, revealing lower activity of this enzyme in breast cancer cells compared to normal cells. The researchers employed a self-immolative trimethyl lock linker design, which connected the fluorophore and the succinate ester, selected as the CES2′s binding moiety. Upon linker release, the ICT mechanism is turned on, resulting in a change in the fluorescence properties (Scheme 15) [53]. Prior to this study, in 2016, Park and other collaborators described the use of similar ratiometric two-photon probes for monitoring CE activities in live hepatocytes and liver tissues [55].

**Scheme 16:**
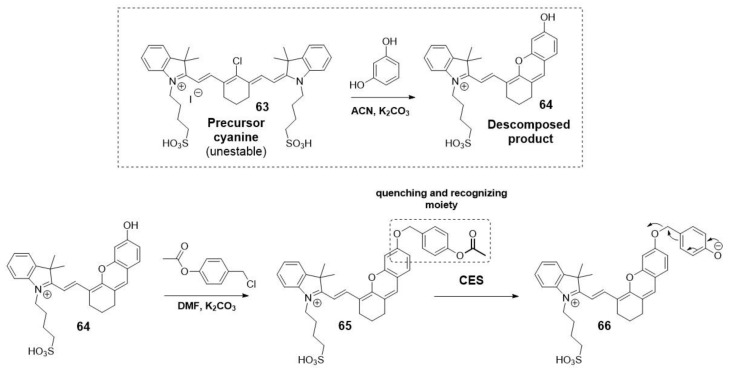
Self-immolative approach for design NIR-fluorescent probe for tracking CES.

Another example used the self-immolative linker 4-(acetoxybenzyl)oxy as both a quenching and recognizing group to develop near-infrared fluorescent probes for tracking endogenous carboxylesterase. Li et al. chose the decomposed fluorophore **64** produced from the unstable cyanine precursor for this NIR fluorescent probe (Scheme 16) [56].

**Scheme 17:**
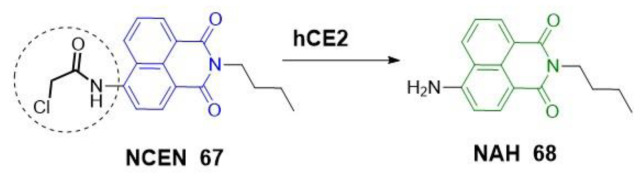
Design of ratiometric fluorescent probe for imaging CES2.

Other fluorogenic scaffolds, such as naphthalimides, have been used by other researchers. Jin et al. designed a two-photon ratiometric fluorescent probe (**67**) for imaging CES2 in living cells in deep tissues, thereby expanding its potential applications in biological systems (Scheme 17). In this study, they attributed the desirable, red-shifted emission observed after deprotection of the amino group to the strong ICT efficiency [57].

**Scheme 18:**
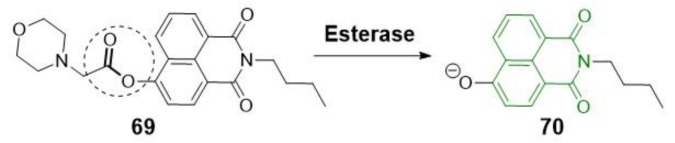
Naphthalimide-derived fluorescent probe for lysosomal detection by ICT monitoring.

Zhang et al. utilized the versatility of the naphthalimide scaffold to incorporate structural modifications, creating a fluorescent probe (**69**) with a morpholine unit for lysosomal binding. By monitoring the ICT after hydrolysis of the carboxylic ester group, the resulting compound (**70**) enabled the assessment of different expression levels of CES2 in both normal and cancerous pancreatic tissues (Scheme 18) [58]. 

**Scheme 19:**
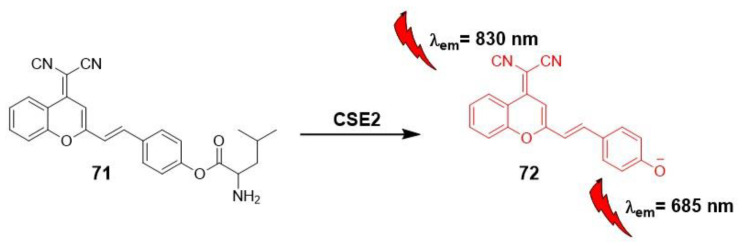
Near-infrared two-photon fluorescent probe for detecting CES2.

In the past two years, the application of carboxylesterase “off-on” fluorescent probes to evaluate their potential as drug activators has gained significant relevance [54]. An illustrative example is presented by Wang et al., who utilized a near-infrared two-photon fluorescent probe, incorporating dicyanomethylene-4*H*-pyran as a precursor scaffold (**71**) for the ICT mechanism (Scheme 19). In their study, Wang et al. used this probe in an orthotopic colon carcinoma mouse model to investigate its effectiveness in detecting carboxylesterase activity and its potential implications as a drug activator.

**Scheme 20:**
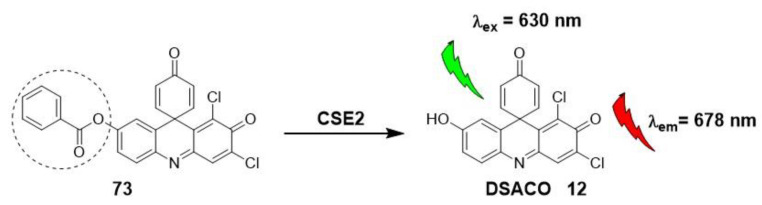
Fluorescent probe based on spiro compounds (DSACO).

The utilization of a near-infrared fluorescent probe **73**, based on spiro compounds and DSACO derivatives, has emerged as a valuable tool for high-throughput screening of herbal medicines (Scheme 20) [52]. 

## 3. PET Technique and PET Probes

Positron emission tomography (PET) relies on different biophysical principles [59,60]. This direct radiolabeling technique finds utility in various fields of nuclear medicine, not only for monitoring pathophysiological processes but also for understanding drug action through tomographic images. The radiotracers used for tagging the cells ex vivo/in vitro, after an incubation period, have an optimal imaging time window. It is necessary to consider this aspect both for their preparation by incorporation of a radioisotope into a biologically active molecule and for the specific tracking studies [61]. 

**Figure 10:**
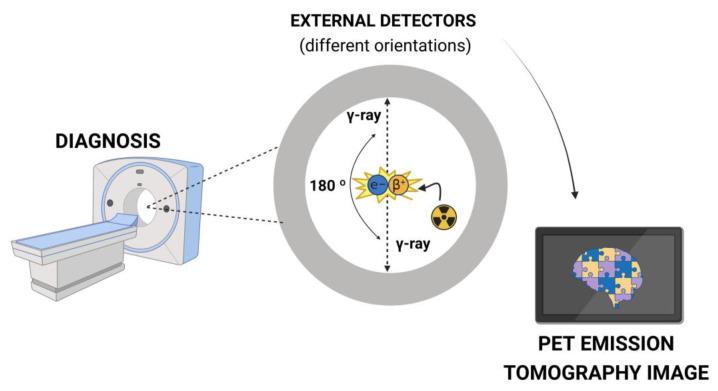
Physical phenomena for diagnosis by PET imaging [61]. Image created using BioRender (www.biorender.com (accessed on 26 July 2023)).

PET imaging agents incorporate a radionuclide in their structure to generate images by detecting the photons generated during the decay process [59]. These radionuclides are isotopes with varying half-lives, such as ^11^C (t_1/2_ = 20.4 min) and ^18^F (t_1/2_ = 109.7 min, the most widely used). They are typically produced in a cyclotron or generator system and emit a positively charged particle known as a positron from the nucleus. This particle travels a short distance within the surrounding tissue before it undergoes annihilation, combining with an electron through a matter-antimatter interaction (Figure 10). The mass of the positron and electron is converted into energy, resulting in the emission of two 511 keV γ-rays (photons) simultaneously, emitted at approximately 180° to each other. The simultaneous registration of radiation allows for the visual and quantitative analysis of biological function [14,59,62].

The spatial resolution of clinical/preclinical PET (6–10 mm) is lower compared to other medical imaging techniques such as magnetic resonance imaging (MRI, 1–2 mm) or computed tomography (CT, 0.1–0.5 mm) [61]. To address this limitation, the combination of PET and CT was introduced in 1994. This led to the development of hybrid systems (PET/CT), revolutionizing neuroimaging by enabling functional and morphological imaging in a single examination [59]. 

### 3.1. Non-Invasive Nuclear Molecular Imaging for Neurological Disorders

Currently, one of the biggest challenges in medicine is understanding the behavior of the human brain. The complexity of the neurological connections, as well as the entirety of receptors, transporters, and neurochemical signaling agents that compose them, remains largely unknown. This lack of knowledge hinders the prognosis and diagnosis of neurodegenerative pathologies [63,64,65].

Moreover, the blood–brain barrier (BBB) plays a crucial role in maintaining the homeostasis of the central nervous system (CNS) and protecting it from invasive and harmful substances [66]. This hampers the adjustment of an appropriate dosage and the penetration of drug-like molecules into the human brain [67]. This factor, combined with the lack of correlation between in vitro and in vivo pharmacokinetic data in conventional predictive models, makes it difficult to reach therapeutic concentrations in the brain and often leads to failures in the discovery process of new chemical entities [68]. 

In this regard, the development of nuclear medicine and new imaging techniques, such as PET, has been an expanding area in preclinical and clinical investigations, as well as in the development of new drugs for both diagnosis and treatment, since the 1980s [69]. Their ability to map various brain targets and neurochemical functions in vivo with practically unlimited penetration capacity, excellent selectivity, and real-time, non-invasive detection has made them a major advancement in neuropsychopharmacology [14,70]. Moreover, only microdoses of radiotracer are required for cell labeling and tracking, as final biological concentrations typically range from nM to pM [71]. Prior to the development of these techniques, traditional invasive methods could not be used directly on the human brain due to ethical reasons and technical complexity. Therefore, most biological information was obtained through *post*-mortem analysis [72].

**Figure 11:**
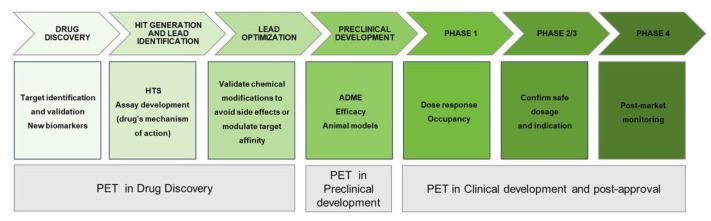
Implications of PET radiotracers in drug discovery [70].

In this sense, PET micro-dose or dose finding studies have indeed emerged as a validated strategy [73]. A radiolabeled drug with high specific radioactivity (˃1 Ci/µm) is administered to trace anatomical distribution and binding affinities to different organs and tissues over time. This approach can be applied either in the early stages of drug candidate selection or even to determine an appropriate clinical dose for an investigational drug (Figure 11) [74].

**Figure 12:**
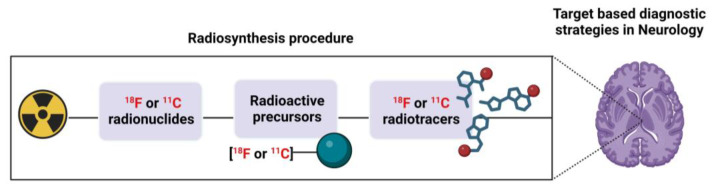
Target based PET diagnosis. Image created using BioRender (www.biorender.com (accessed on 26 July 2023)).

For example, PET radiopharmaceuticals provide important and valuable knowledge in various aspects of drug development and clinical use [75]. Firstly, they offer insights into the pharmacokinetics and distribution of a drug during the early stages of drug development. Secondly, PET imaging provides pharmacodynamic information, which allows for determining the relationship between the neurochemical and cognitive/behavioral effects of the drug. This aids in assessing the drugs therapeutic potential. Thirdly, PET enables the monitoring of therapeutic responses during clinical use, providing valuable data on the drugs effectiveness. Lastly, PET imaging can aid in the identification of new biomarkers and their involvement in pathological processes. These aspects are vital for the success of a drug candidate during clinical trials (Figure 12). For this reason, the use of PET imaging targeting the CNS is considered a promising tool in both preclinical and clinical processes [76]. The output of PET imaging depends on the chemical nature of the radiotracer and the physiological conditions of the living subjects. Thus, the design of a specific radiotracer is an essential step to achieve target selectivity and appropriate distribution in the region of interest (Figure 12) [77,78]. 

**Figure 13:**
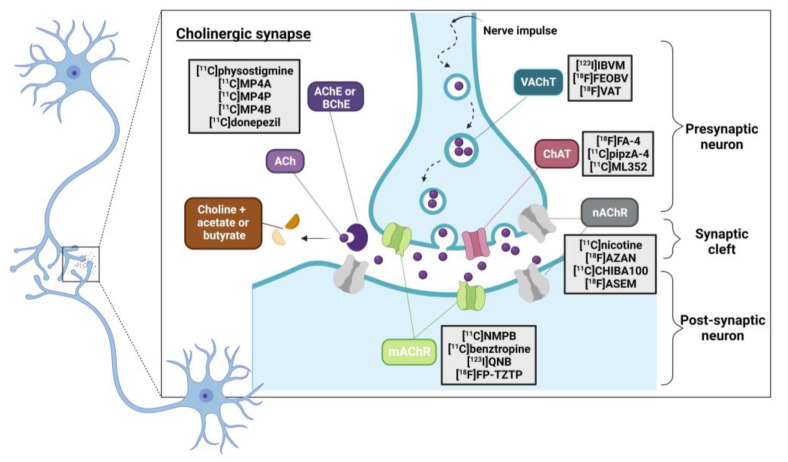
PET probes targeting the cholinergic system [12,79]. Image created using BioRender (www.biorender.com (accessed on 26 July 2023) based on Reprinted (adapted) with permission from [79]. Copyright 2021, American Chemical Society.

One of the essential approaches in the investigation of neurodegenerative disorders is the examination of changes occurring at the neuronal metabolism level. In this regard, the use of PET is essential for studying the balance between transmitters and receptors in brain systems such as dopaminergic, serotonergic, or cholinergic systems. Within the neurotransmitter pathway, the presynaptic neuron, the postsynaptic neuron, and the intraneuronal metabolism are the typical locations of interest (Figure 13). Specifically, the neuroimaging of the central cholinergic system in the CNS has been a rapidly growing field since the 1980s [79]. This complex and multi-component neurotransmitter system uses acetylcholine for the transduction of action potentials during cholinergic synapses [12] and plays a crucial role in brain functions such as attention, memory, emotions, cognition, and consciousness. 

Several studies have emphasized the use of radioligand imaging agents to target different components of the cholinergic system involved in neurodegenerative diseases such as dementia, particularly Alzheimer’s disease (AD), which accounts for 60–80% of cases [80,81]. Other frequently encountered disorders include frontotemporal dementia (FTD) and dementia with Lewy bodies (DLB). 

These targets are usually categorized based on their function, including acetylcholine receptors such as nicotinic (nAChRs) and muscarinic (mAChRs); neurotransmitters such as acetylcholine (Ach); transporters like vesicular acetylcholine (VAChT); and presynaptic high-affinity choline uptake transporters (CHT-1) as well as enzymes including choline acetyltransferase (ChAT), acetylcholinesterase (AChE), and butyrylcholinesterase (BuChE) [79,82].

#### 3.1.1. PET Probes for Imaging AChE and BChE in Dementia Disorders

In the context of this review, we will focus on two major esterase enzymes involved in the cholinergic system, AChE and BuChE, for in vivo nuclear imaging. These hydrolytic enzymes catalyze the breakdown of cholinergic esters into choline and the corresponding acetate or butyrate ions. They play a regulatory role in maintaining appropriate levels of acetylcholine (Ach) in the synapse [83]. Dysregulation of their activities has been linked to different neurogenerative disorders, including AD, which is characterized by severe progressive cognitive and motor impairment. The cholinergic dysfunction hypothesis [84] has been considered, along with the deposition of extracellular misfolded beta-amyloid (senile plaques) and intraneuronal τ-protein aggregation (neurofibrillary tangles), as one of the crucial causes of this chronic, multifactorial disease, but to date, of unknown etiology [85,86]. This hypothesis is supported by previous observations of reduced cholinergic activity, particularly ACh levels, in *post-mortem* autopsies of AD brain patients.

In this respect, the implications of AChE and BuChE are well accepted. It has been reported that the presence of AChE is significantly reduced in the brains of late-stage AD patients (90%), whereas BuChE activity remains unchanged or even increases progressively. This suggests that BuChE may have a compensatory role in maintaining ACh levels [87,88]. In addition, several studies provide evidence that these cholinesterases (ChEs) are also involved in secondary neuropathogenic phenomena, such as the processing and deposition of senile plaques [88]. Other studies link their contribution to other diseases, including cardiovascular pathologies, obesity, diabetes mellitus type 2, or even cancer [87]. 

As a result of their involvement in AD and related dementias, ongoing research is focused on the development of new promising treatments and early diagnostic methods. Currently, cholinesterase inhibitors such as Donepezil (Aricep^®^), Rivastigmine (Exelon^®^) or Galantamine (Razadyne^®^) are some of the few drug therapies that have clinically demonstrated the ability to temporarily minimize and/or stabilize symptoms at different disease stages [89]. They share a basic mechanism of action, which consists of the inhibition of ChEs to prevent the breakdown of Ach. 

**Figure 14:**
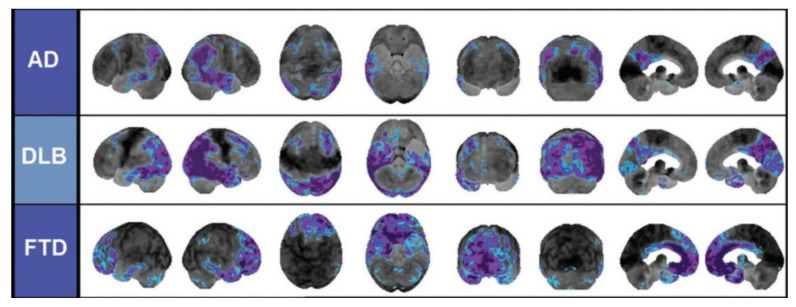
PET radiotracer to distinguish neurodegenerative disorders (asymmetric altered pattern) [90]. Reprinted with permission from [90]. Copyright 2014, the Radiological Society of North America (RSNA^®^).

In this context, the use of PET radiotracers that target AChE and/or BuChE can be considered a promising strategy to facilitate the development of effective treatments or monitor therapy response. By accurately designing these radiotracers with the aid of computational techniques and taking advantage of their affinity and target selectivity, they can be used to differentiate between different neurodegenerative disorders (Figure 14) [90].

Since the late 1990s, several investigations have been conducted in AD using two different types of AChE’s PET radioprobes. A series of reversible or irreversible AChE inhibitors (AChEIs) have been labeled with ^11^C and ^18^F to map and visualize AChE binding sites in the brain. This pioneering strategy, which uses AChEIs themselves as radiotracers, enables not only a mapping of active sites or enzyme activity in different brain regions but also a direct analysis of the pharmacokinetic properties of these AChEIs. Another strategy involves the design of labeled analogues of acetylcholine to quantify AChE activity by measuring trapped membrane-impermeable polar metabolites that result from the hydrolysis of these analogues. This approach allows for the measurement of changes associated with aberrant enzyme activity [91].

##### First Class of AChE’s PET Probes (Labeled AChEIs)

**Scheme 21:**
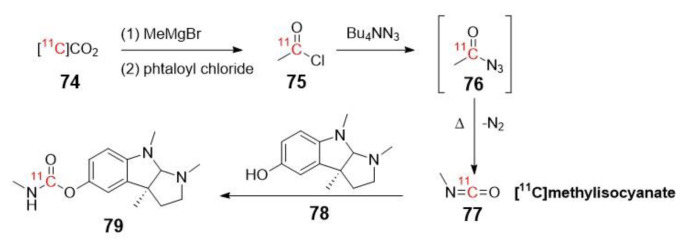
Radiosynthesis of [^11^C]-Physostigmine via nucleophilic attack of the [^11^C]methylisocyanate [79].

In 1996, Pappata et al. reported the first in vivo imaging study of human AChE distribution at different anatomical cerebral levels (striatum ˃ cerebellum ˃ thalamus ˃ cerebral cortex) using [^11^C]Physostigmine (**79**) as a tracer [92]. The synthesis of this radiolabeled AChE inhibitor was previously described by Bonnot-Lours et al. in 1993 (Scheme 21) [93].

**Scheme 22:**
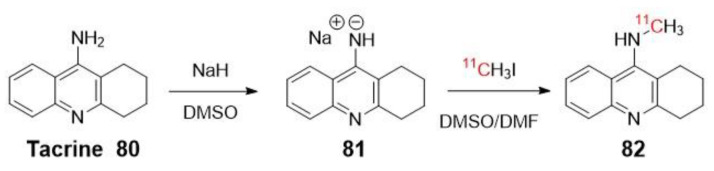
Radiosynthesis of [^11^C]methyltacrine via nucleophilic attack.

Previously, in 1991, the same group reported *N*-[^11^C]methyltacrine (**82**) as a radioligand (Scheme 22) [94]. However, this tracer exhibited non-specific binding to brain regions, which could be attributed to the lack of selectivity of AChE over BuChE [92,95]. 

**Scheme 23:**
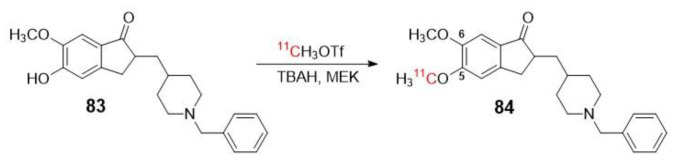
Radiosynthesis of [^11^C]-donepezil **84 [97]**.

Funaki et al. described in 2003 the radiosynthesis protocol for [5-^11^C-methoxy]-donepezil ([^11^C]-donepezil) **84** (Scheme 23), a representative inhibitor radiolabeled with an *N*-benzylpiperidine moiety (**83**) that was examined in vitro and in vivo in rat brain [96]. The study proposed the use of **84** for in vivo visualization of AChE in the human brain and for evaluating the efficacy of AChE inhibitor therapies [96]. In 2007, Okamura et al. utilized **84** for PET imaging to measure in vivo AChE density in the brains of patients with AD following 6-month oral administration of donepezil [97]. The aim of this study was to validate **84** as a tool for the pharmacological evaluation of donepezil.

Previously, De Vos et al. reported the biological evaluation of [^11^C]-donepezil as a radiotracer for studying AChE, but with the difference that the methoxy group was the one in position 6 [98]. This study did not yield conclusive results regarding the distribution of AChE in the brain. Nevertheless, it highlighted the importance of radiolabeling at the appropriate position of the structure [97]. 

Only a few examples in the literature use ^18^F for labeling molecules as potential radiotracers to measure acetylcholinesterase in the brain, despite its several advantages over ^11^C. One such advantage is its ease of cyclotron production and longer half-life (109.8 min vs. 20.4 min), which allows for longer periods of in vivo scanning of biological processes, providing more time to study and observe the desired phenomenon. Finally, from a drug development perspective, the use of fluorine as a bioisostere of hydrogen offers convenience due to its small van der Waals radius, strong bonding capability with carbon, high electronegativity, and lipophilicity [88,99,100].

**Scheme 24:**
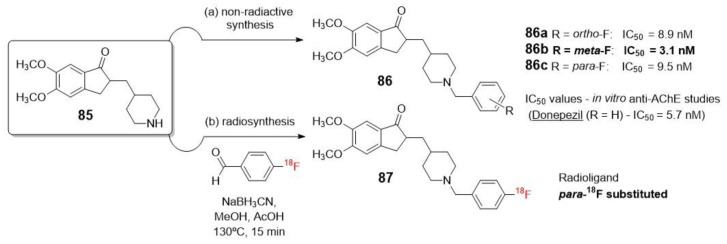
Feasible obtention of a potential non-radioactive drug candidate and radiosynthesis of [^18^F]4 (para-substituted) isomer.

One attractive example of using ^18^F radionucleotides is the work of Lee et al., who described the synthesis and biological evaluation of halogen-substituted donepezil analogues, including their radiolabeled forms. This research highlights the importance of a drug candidate that not only exhibits good activity but also allows for a non-radioactive synthesis that can be adapted to a feasible radiosynthesis within a short period of time and with good yields [101]. In this case, although the presence of an unlabeled fluorine in the C-3 position of the phenyl ring (**86b**) demonstrated the highest AChE inhibitory activity, the [^18^F]fluorination at the less reactive *meta*-position was limited by labeling methods (Scheme 24). Therefore, they ultimately opted to prepare the C-4 (*para*-^18^F substituted) analogue of donepezil (**87**) through a reductive amination with ^18^F-labeled benzaldehydes, which was previously reported by Wuest (Scheme 24) [100].

**Scheme 25:**
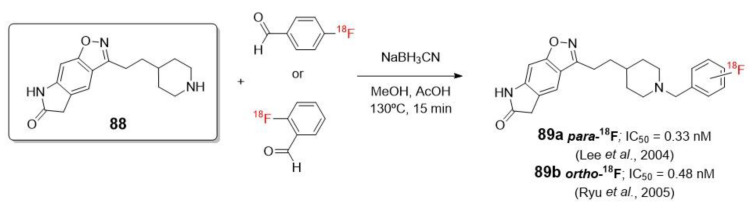
Radiosynthesis of [^18^F]2 (ortho) **89a** and [^18^F]4 (para) **89b** with *N*-benzyl piperidine lactam benzoisoxazole moiety as a pharmacophore [102,103].

The same conclusion was also observed in the independent works of Lee [102] and Ryu [103], who studied a series of ^18^F labeled compounds that contain the *N*-benzyl piperidine benzoisoxazole lactam moiety as a pharmacophore (**89a**–**b**, Scheme 25).

**Figure 15:**
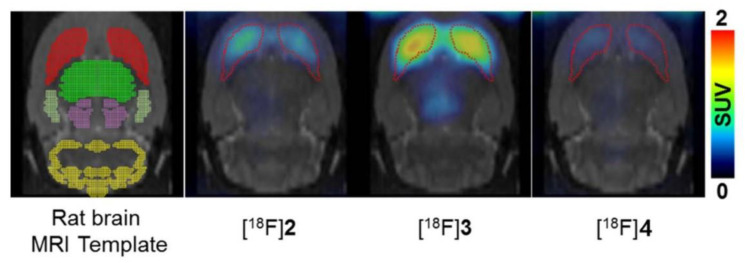
PET/CT images comparing the different [^18^F] isomers ([^18^F]2 is ortho, [^18^F]3 is meta and [^18^F]4 is para-substituted) in a normal rat brain [104]. Reprinted from publication [104], Copyright 2017, with permission from Elsevier.

As a continuation of these results and with the advancement of new radiochemical methodologies, Lee et al. recently synthesized the ^18^F labeled *meta*-isomer ([^18^F]3) of CP-118,954 using diaryliodonium salt precursors for direct nucleophilic ^18^F-labeling. Their aim was to evaluate the in vivo affinity of AChE in rat brains, and their findings demonstrated that AChE’s affinity is influenced by the position of aromatic fluorine, as represented in Figure 15 [104].

**Scheme 26:**
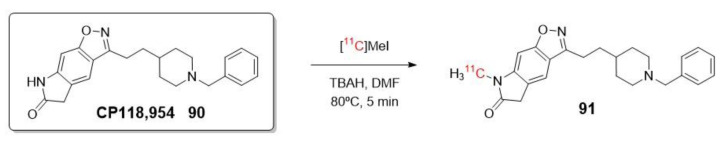
Radiosynthesis of **91**.

Using the same pharmacophore as Lee and Ryu, in 2002, Musachio et al. reported the radiosynthesis of CP118,954 (**90**) labeled with ^11^C instead of ^18^F (Scheme 26) [105]. In parallel, Bencherif et al. conducted a study of this inhibitor as a PET imaging agent to demonstrate the response to AChEIs such as donepezil and to assess changes in AChE binding sites during the progression of AD [106].

**Figure 16:**
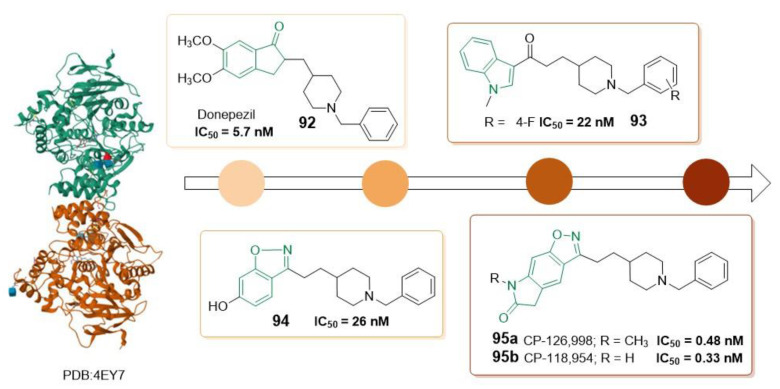
Types of pharmacophores to design PET probes for AChE (PDB: 4EY7) based on different inhibitors.

This type of inhibitor, benzoisoxazole lactam derivative **95**, demonstrated potent in vitro enzyme inhibition in the sub-nanomolar range compared to other related AChE inhibitors containing *N*-benzylpiperidine with indanone (**92**) [98], benzoisoxazoles (**94**) [107], or indoles (**93**) [108] groups (Figure 16). Most of these compounds also exhibited high selectivity for AChE over BuChE. However, despite the strong binding properties and enzymatic inhibitory activity in the nanomolar range of **92**, **93**, and **94**, these inhibitors failed in vivo mapping of AChE. The lack of correlation between the radioactivity of these PET probes and the AChE binding affinity values, as well as previous data reported about the density of AChE in the brain, shows the importance of selecting a specific pharmacophore to design a suitable radiotracer with proper biodistribution [106].

**Figure 17:**
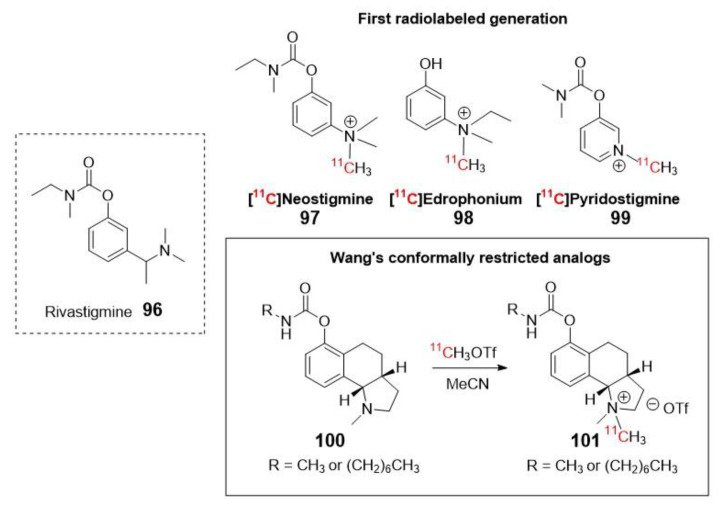
First generation of radiolabeled rivastigmine (tertiary amine) and radiosynthesis of new conformationally restricted quaternary ammonium rivastigmine analogues.

Wang et al. developed a ^11^C-radiosynthesis method for conformationally restricted quaternary ammonium rivastigmine analogues to image both AChE and BuChE (Figure 17, **101**), based on a newer generator inhibitor with dual activity on both enzymes. These probes, which belong to a different class of enzyme inhibitors, exhibit higher affinity compared to their tertiary amine precursors. However, the presence of a positive charge in their structure hampers their ability to pass through the BBB. Therefore, the authors proposed their potential application as tracers for cardiac imaging of AChE and BuChE. This highlights the importance of designing drugs/tracers that can effectively cross the BBB when targeting the CNS in neurodegenerative disorders [109].

**Scheme 27:**
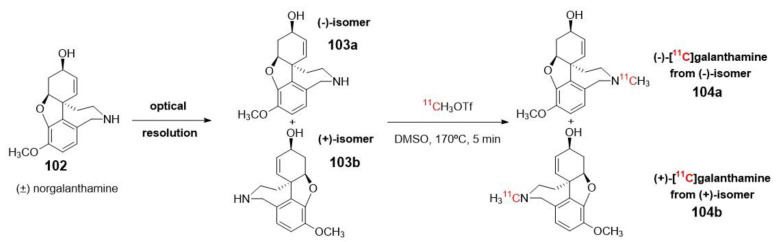
Obtention and radiolabeling of (−)- and (+)-galanthamines.

Optically pure (−)-galanthamine, another traditional AChE inhibitor that has received clinical approval for the treatment of mild to moderate dementia in patients with AD [110], is also used in the development of potential radiopharmaceutical probes for imaging brain AChE. Building upon this inhibitor, in 2014, Kimura et al. described the synthesis and radiolabeling of (−)- and (+)-galanthamines (**104a**–**b**) by *N*-methylation using [^11^C]methyl triflate of norgalanthamines. These precursors were obtained optically pure by chiral resolution (Scheme 27) [111].

In vitro and in vivo experiments were conducted using these compounds in mice to study the distribution and activity of AChE. Biodistribution studies revealed significant differences in the accumulation of radioactivity in different brain sections (such as the striatum and cerebellum) for both tracers. Furthermore, a different response to pre-treatment with donepezil was also observed in blocking experiments (Scheme 27). These findings demonstrated that only (−)-[^11^C]galanthamine **104a** can serve as a PET tracer for imaging regions with abundant AChE, providing insights into the pathogenesis and progression of AD. This can be attributed to its similar AChE inhibitory activity to commercially available (−)-galanthamine hydrobromide, along with its specific binding properties [111]. 

**Figure 18:**
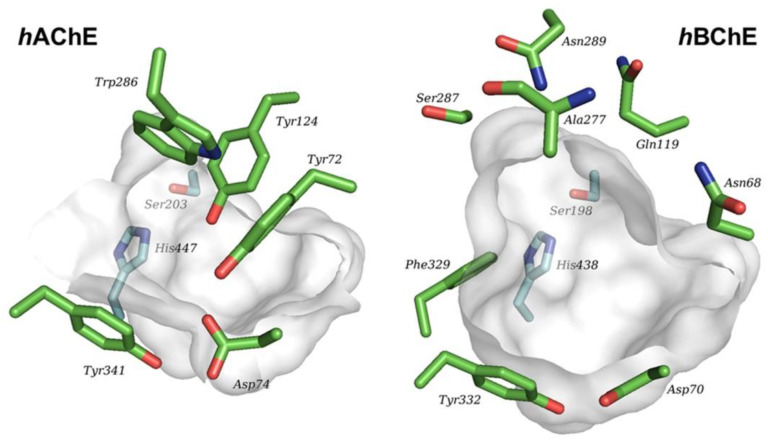
Pymol comparison of hAChE (PDB 4EY4) and hBChE (PDB 6QAA) catalytic sites [114]. Reprinted with permission from [114] Copyright 2021, American Chemical Society.

Recent studies have focused on the design of PET probes with enhanced selectivity between AChE and BuChE. Despite sharing 65% of the aminoacid sequence, these two enzymes differ in their tissue distribution, kinetic properties, and substrate specificity. AChE is predominantly found in nerve cells, specifically in the synaptic cleft (in its soluble form) and in the synaptic membranes (in its bound form). On the other hand, BuChE is primarily associated with glial cells [112]. Both enzymes present a catalytic active site situated at the bottom of a hydrophobic gorge as well as a peripheral anionic site. However, the gorge volume of the catalytic site in BuChE is significantly larger (~200 Å) compared to that of AChE (Figure 18) [113]. This conformational disparity enables BuChE to accommodate larger substrates and confers differences in their substrate specificity [112].

**Scheme 28:**
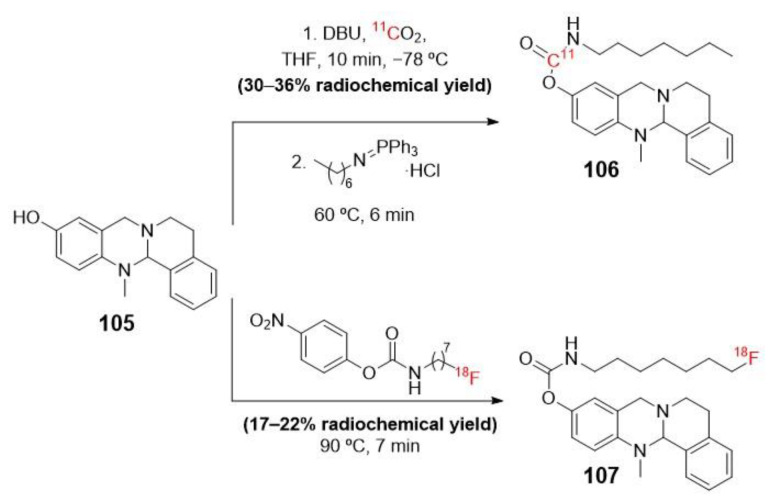
Synthetic routes for preparing a selective radiolabeled pseudo-irreversible BuChE PET probe.

In this context, Sawatzky et al. were the pioneers in synthesizing a selective and potent inhibitor-type radiotracer for BuChE. They achieved this by incorporating ^11^C or ^18^F as radioisotopes into the carbamate moiety (**105**) of a tetracyclic precursor (Scheme 28) [115].

**Figure 19:**
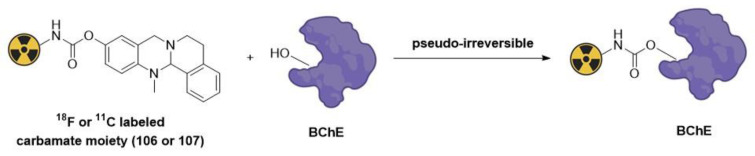
Mechanism of action of pseudo-irreversible PET probes via carbamoylation.

As shown in Figure 19, the mechanism of action of these PET probes is based on covalent and pseudo-irreversible radiolabeling through a carbamoylation reaction of a serine hydroxyl residue at the active site of the BuChE enzyme [71].

In vitro kinetics of enzyme inhibition and a first ex vivo autoradiography with healthy mouse brain slices were also carried out to demonstrate that this type of tracer could enable the in vivo mapping of BChE distribution [115].

The inhibition of this type of ligand is transient due to the chemical instability of carbamates. This fact hinders the design of these carbamate-based PET tracers due to the complexity of their kinetic and binding behaviors. It is also necessary to achieve a precise balance in the carbamoylation rate to accurately reflect the true distribution of BuChE throughout the body and the CNS [87]. Several publications have reported that the introduction of heterocyclic moieties and polar groups at the end of alkyl chains in these carbamate inhibitors might improve physicochemical properties, such as protein binding, penetration through the BBB, and water solubility [114].

**Scheme 29:**
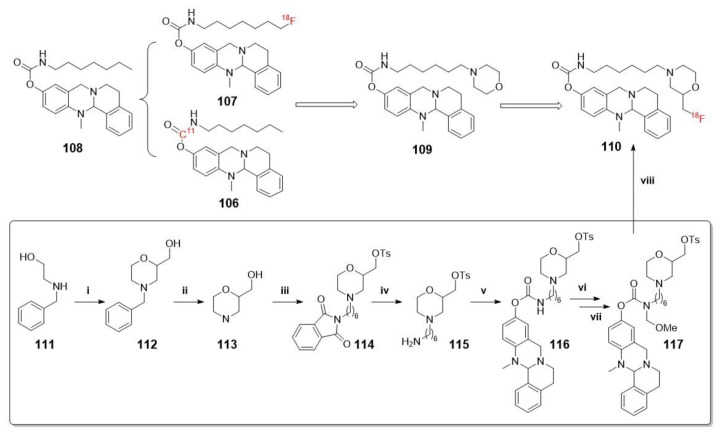
Synthesis of **110**. Reagents and conditions: (**i**) 1. epichlorohydrine, RT, 3 h; 2. H_2_SO_4_, 140 °C, 1 h; 3. H_2_O, CHONH_2_, 145 °C, 20 h; (**ii**) H_2_, Pd/C, MeOH, RT, 1 h; (**iii**) 1. 2-(6-bromohexyl)isoindoline-1,3-dione, *N*,*N*-diisopropylethylamine (DIPEA), DMF, 100 °C, 20 h; 2. TosCl, NEt_3_, CH_2_Cl_2_, RT, 20 h; (**iv**) H_4_N_2_·H_2_O, EtOH, 80 °C, 1.5 h; (**v**) 1. p-nitrophenyl chloroformate, NEt_3_, CH_2_Cl_2_, 3 h, RT; 2. 13-methyl-5,8,13,13a-tetrahydro-6H-isoquinolino [1,2-b]quinazolin-10-ol, NaH, CH_2_Cl_2_, 2 h, RT; (**vi**) TMS Cl, para-formaldehyde, CH_2_Cl_2_, RT, 18 h; (**vii**) MeOH, RT, 1 h; (**viii**) 1. [ ^18^F]KF, K_222_, MeCN, 110 °C, 10 min; 2. 6 M HClaq, 90 °C, 5 min.

For this reason, more recently, Gentzsch et al. have developed a new generation of ^18^F-PET carbamate tracers with a morpholine moiety at the end of alkyl chains, leading to a significantly prolonged duration of action. The synthesis of these probes has been carried out using a novel protecting group strategy for ^18^F radiolabeling of carbamate precursors (Scheme 29) [87].

##### Second Class of AChE´s PET Probes (Analogues of ACh)

**Figure 20:**
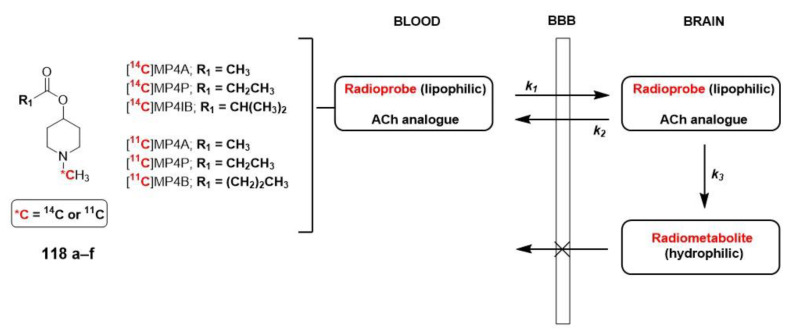
Examples of substrate-type radioprobes and kinetic model representation.

The design of substrate types for AChE and/or BuChE radioprobes for in vivo PET imaging is based on modifying the structure of ACh to obtain a neutral, lipophilic substrate that is permeable to the BBB. As depicted in Figure 20, once the radioprobe (of a lipophilic nature) enters the brain (*k*_1_), it undergoes hydrolysis to form a polar radiometabolite that is unable to cross the membrane (due to its hydrophilic nature). The ratio of the trapped radiometabolite relies on the activities of ChEs (*k*_3_), and the evaluation of the generated radioactivity is crucial in elucidating the role of cholinesterase enzymes in AD. According to Kikuchi et al., when applying the two-tissue compartment kinetic model, it is also important to consider the rate of back diffusion of the radioprobe (*k*_2_) and the rate of metabolite elimination (*k_el_*) to obtain an accurate value for *k_3_*, which is associated with AChE and/or BuChE activities [91].

In most of the examples described in the literature, the basic scaffold consists of a *N*-methylpiperidin-4-yl ester with a different acyl group (acetate, propionate, isobutyrate, and butyrate), which is hydrolyzed to an *N*-methylpiperidin-4-ol metabolite. This acyl group determines the specificity of the enzyme. As mentioned earlier, larger substrate sizes provide a better fit for BuChE. Hence, [^11^C]MP4A (**118d**) and [^11^C]MP4P (**118e**) exhibit higher specificity for AChE compared to BuChE, whereas [^11^C]MP4B (**118f**) is considered a potential radiopharmaceutical for BuChE. Currently, the first two ^11^C labeled analogues are approved for clinical use in neurodegenerative diseases by PET (Table 1) [116,117].

For instance, in a small clinical two-phase study lasting 12 months, Kadir et al. utilized [^11^C]MP4P (**118e**) to investigate the effect of galantamine on cortical AChE and nicotinic receptor binding in 18 patients with mild AD. The primary focus of the study was to evaluate these effects using PET imaging techniques [118].

**Figure 21:**
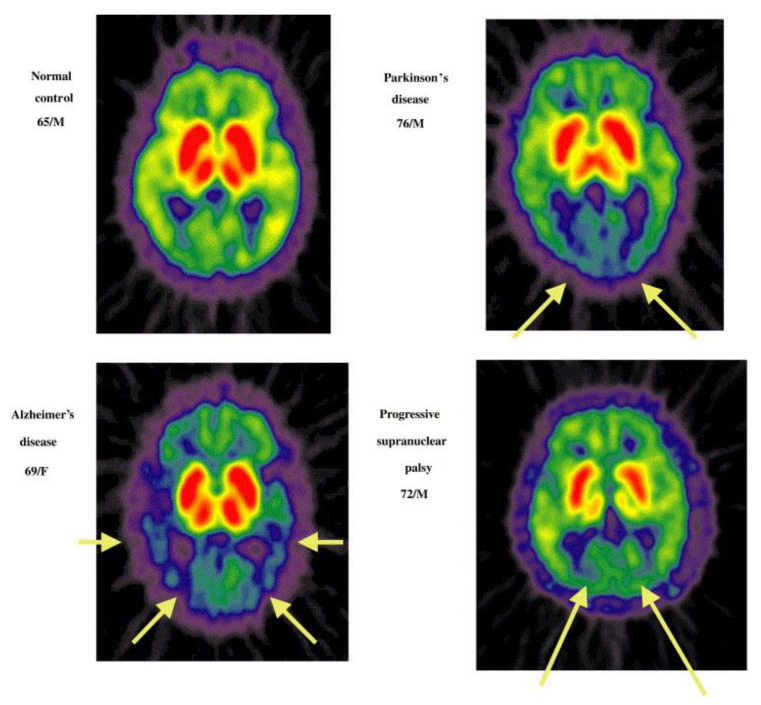
PET images of brain patients with different neurodegenerative disorders after injection of **118d**. Reprinted with permission from [119] Copyright 2002 Elservier.

As mentioned in the introduction, authors such as Namba et al. and Shinotoh et al. have proven the use of these radioligands as a valid strategy to distinguish between AD (B), Parkinson’s disease (C), and progressive nuclear palsy (D). This distinction is supported by the PET-generated images shown in Figure 21 [119,120].

The design of PET probes with *N*-methylpiperidin-3-yl esters has also been studied. However, the presence of an asymmetric carbon in these compounds complicates their use as radiotracers. Prior separation of the optical isomers is necessary to establish a proper correlation with the described kinetic model. This separation is required due to the different rates of hydrolysis of isomers by AChE, and it is essential for the accurate design of a radioprobe [121].

**Scheme 30:**
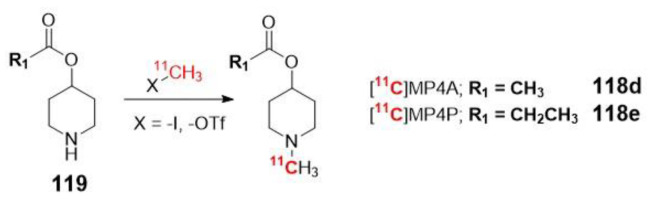
Most commonly method for radiosynthesis of ^11^C-radiolabeled ACh analogues.

In terms of the radiosynthesis of these probes, the most commonly used method is *N*-[^11^C]methylation using [^11^C]methyl iodide or [11C]methyl triflate as radiolabeled precursors. However, when mapping cerebral AChE using the metabolite trapped method, it is not suitable to include the radioisotope in the acyl group. If the acyl group were radiolabeled, the resulting [^11^C]acetic acid generated in the blood would enter the brain and be metabolized by glial cells into ^11^CO_2_, which would be rapidly cleared from the brain (Scheme 30) [91].

**Scheme 31:**
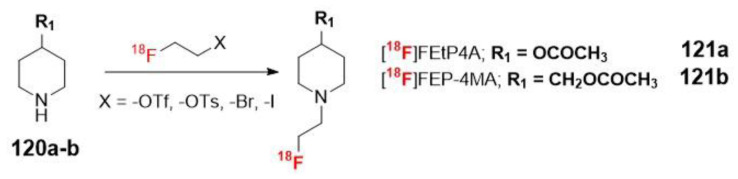
Radiosynthesis of ACh ^18^F-anologue substrate type PET probes.

Once again, ^18^F radiolabeled derivatives were designed to increase the half-life of the radiotracer. The most significant examples are [^18^F]FEtP4A (**121a**) and [^18^F]FEP-4MA (**121b**, Scheme 31). However, the preparation strategy had to be modified for these tracers due to the instability of the fluoromethyl group attached to the secondary amine and the adverse effect on AChE activity caused by the undesirable fluorine ion generated through defluorination. Kikuchi et al. described the successful preparation of these radioligands through the use of radioactive fluoroethylation of *N*-piperidin-4-yl esters (**120a-b**) with [^18^F]fluoroethyl triflate, tosylate, or different halogens (-Br, -I) [91].

## 4. Materials and Methods

The bibliographic search was carried out using the Google and Google Scholar search engines combined with different databases such as PubMed (https://pubmed.ncbi.nlm.nih.gov (accessed on 1 April– 26 July 2023)), Web of Science (www.webofscience.com (accessed on 1 April–26 July 2023)), or Scopus (www.scopus.com (accessed on 1 April–26 July 2023)). All the chemical structures were drawn using ChemDraw 22 (www.perkinelmer.com (accessed on 1 May–17 August 2023)). The graphical abstract representation and the indicated figures were created using BioRender (www.biorender.com (accessed on 1 July–23 August 2023)).

## 5. Conclusions

While there are several reviews in the literature that compile probes targeting enzymes, very few encompass esterases, and, to the best of our knowledge, none have been published in the last two years. This is why we find it relevant to gather PET and fluorescent probes that target esterases, focusing mainly on acetylcholinesterases, butyrylcholinesterases, and carboxyesterases. These enzymes are involved in pathologies of the central nervous system and cancer, which have a high incidence in the population.

The development of probes targeting these enzymes is of utmost importance, not only for enabling early diagnosis to improve patients’ prognosis but also for aiding in therapy monitoring and even the development of new drugs. Additionally, the advancement of new non-invasive in vivo imaging approaches has broad applicability, operating at various levels, including molecular, cellular, and organ levels, or even the whole organism.

In conducting this review, we focused on analyzing the probes developed for different applications and drawing conclusions about the key factors to consider during probe development. While our review is centered on esterases, the methodologies employed and the conclusions reached can be applied to probes targeting any other target for the aforementioned diseases or others. We believe that sharing this knowledge will be beneficial to the scientific community interested in entering this area of research. In fact, within our research group, we have embarked on a new line of investigation to develop PET and fluorescent probes targeting kinases using this knowledge.

## Figures and Tables

**Table 1:** Properties of most widely used ^11^C radiotracers clinically approved to evaluate neurodegenerative disorders [117].

	Selectivity for AChE		
Radiotracer	Human cerebral cortex (Irie et al. 1996)	PET scan time	Optimum brain regions for measurement in humans
[^11^C]MP4A	94%	40–60 min	Cerebral cortex, thalamus
[^11^C]MP4P	86%	60–80 min	Cerebral cortex, thalamus, and cerebellar cortex (striatum)

## Data Availability

Not applicable.
